# Oncoplastic Reduction Pattern Technique Following Removal of Giant Fibroadenoma

**Published:** 2018-01-22

**Authors:** Andrea Hiller, Thomas J. Lee, Joshua Henderson, Nicolas Ajkay, Bradon J. Wilhelmi

**Affiliations:** ^a^School of Medicine, University of Louisville, Louisville, Ky; ^b^Division of Plastic and Reconstructive Surgery; ^c^Division of Surgical Oncology, Department of Surgery, University of Louisville, Louisville, Ky

**Keywords:** giant fibroadenoma, oncoplastic, reconstruction, breast, Wise-pattern reduction

## Abstract

**Objective:** Oncoplastic surgery was developed to allow for large tumor excision and immediate breast reconstruction with the goal of optimal breast shape and symmetry. Although initially used in women who underwent lumpectomy for breast malignancy, these techniques can be useful for cosmetic issues caused by benign breast disease. We describe a modification of an inferior pedicle with Wise-pattern reduction mammoplasty for oncoplastic reconstruction of a giant fibroadenoma. **Methods:** A 30-year-old woman with size 32 DD breasts was referred by the surgical oncologist with a biopsy-proven fibroadenoma of the right breast. Surgical oncology excised the mass, and immediate reconstruction was performed with an inferolateral pedicle Wise-pattern reduction technique. **Results:** Immediately postoperatively, the patient showed excellent symmetry. Follow-up postoperatively showed good wound healing, preserved symmetry, and a viable, sensate nipple. **Conclusions:** Oncoplastic breast reconstruction in a reduction pattern technique after giant fibroadenoma removal provides an excellent outcome, allowing for improved symmetry.

Fibroademonas are benign breast neoplasms that arise from the epithelium and stroma of the terminal-duct lobular unit.^1^ These lesions are common and are typically found in premenopausal women aged 20 to 40 years but can be seen in women of any age. Most fibroadenomas present as a distinct, painless breast mass that is discovered by the patient and are usually less than 3 cm in size.^2^ On occasion, the tumor may exceed 5 cm and is classified as giant fibroadenoma.^3^^-^5

The usual recommendation for masses found to be consistent with fibroadenomas on physical examination and imaging is observation.^4^ Although benign, giant fibroadenomas are often treated with surgical excision due to patient discomfort, back pain, progressive growth, and aesthetic concerns.^3^ It may also be necessary to rule out other forms of malignancy, as both benign and malignant phyllodes tumors can mimic the presentation of fibroadenomas.^2^^,^^3^

Although excision is the standard treatment of giant fibroadenomas, the extent of surgery is somewhat controversial and varies from excisional biopsy to reduction mammoplasty or even subcutaneous mastectomy.^4^^,^^6^ Simple excision may be satisfactory for patients with small- to moderate-sized lesions; however, those with larger tumors can be left with a displeasing, loose, ptotic breast and may require secondary surgery to address these issues.^7^

Oncoplastic surgery has become increasingly popular in recent years, combining the principles of oncology and plastic surgery with the goal of obtaining maximal tumor resection and optimal cosmesis in women who require lumpectomy for breast cancer.^8^^,^^9^ Oncoplastic techniques account for tumor location and size, tumor to breast ratio, and the desires of the patient.^10^ Although giant fibroadenomas are benign lesions, the surgeon is faced with a reconstructive challenge similar to when a large malignant breast neoplasm is removed. There are 2 main principles in oncoplastics: volume displacement and volume replacement.^9^ In this case, a volume displacement technique was chosen.

We present a 30-year-old woman with a giant fibroadenoma, measuring 11.5 cm, in the right breast. After excision of the lesion, reconstruction was accomplished with an inferolateral pedicle Wise-pattern breast reduction approach.

## METHODS

A 30-year-old woman with size 32 DD breasts was referred by the surgical oncologist with core needle biopsy-proven fibroadenoma of the right breast. Physical examination showed gross breast asymmetry, with the right being larger than the left. There was an 11-cm palpable mass in the central quadrant of the right medial breast that extended into the upper and lower quadrants. Because of the size of the mass, it was considered a giant fibroadenoma.

**Figure 1:**
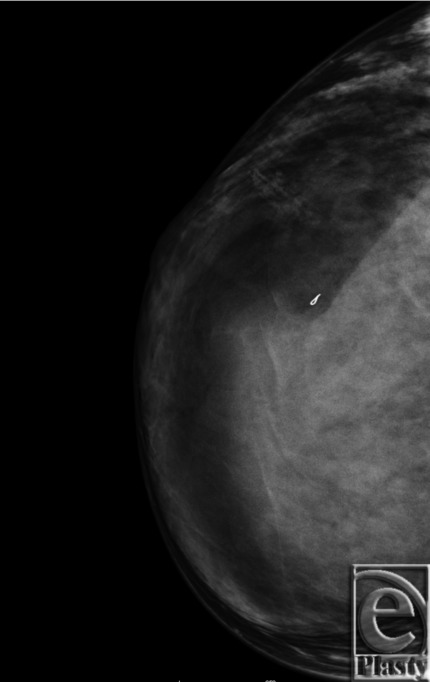
Preoperative mammogram.

Preoperative mammographic studies revealed an isodense, oval mass measuring 11.5 cm, with circumscribed margins in the central region of the right breast (Fig 1). Ultrasound scan showed an oval, circumscribed, mixed echogenicity but predominantly hypoechoic mass measuring 10×3×8 cm. The mass demonstrated some internal color Doppler flow and had central echogenic and anechoic components. Because of the size of the mass and associated symptoms, excision with immediate reconstruction was agreed upon.

**Figure 2:**
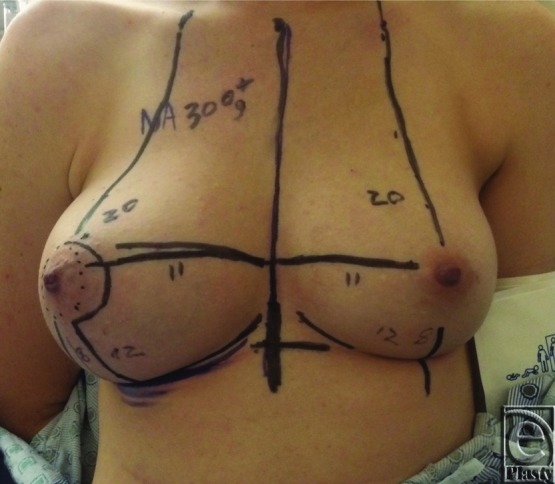
Preoperative markings.

**Figure 3:**
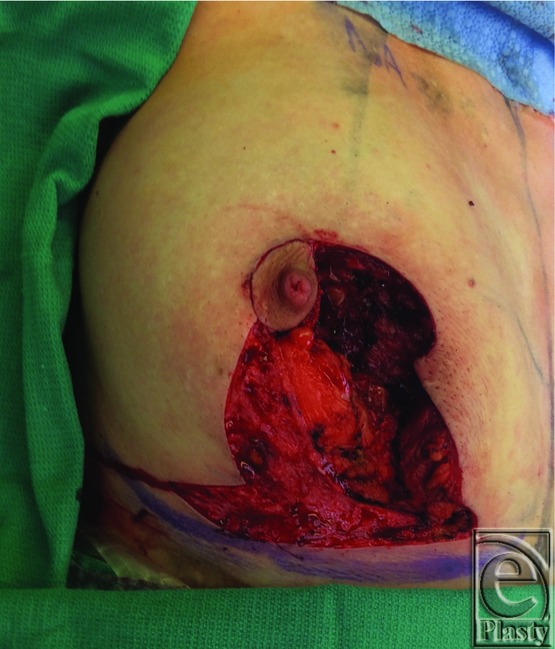
Dermoglandular pedicle.

**Figure 4:**
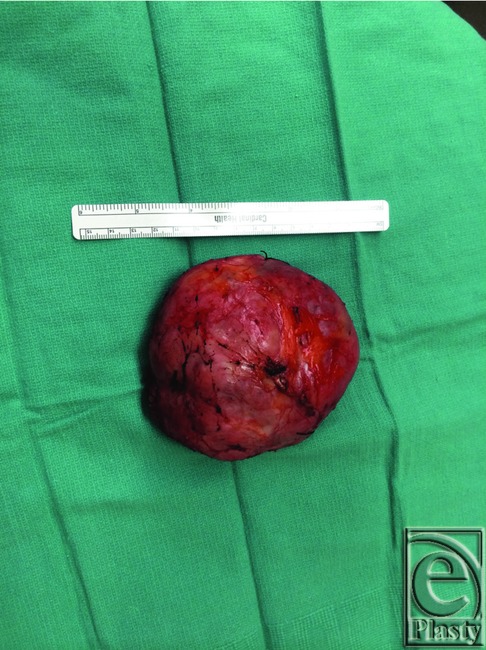
Resected giant fibroadenoma.

Preoperative markings were based on a breast reduction technique using an inferior pedicle with Wise-pattern skin reduction (Fig 2). The right nipple was displaced laterally compared with the left, and skin excision was planned to reposition the nipple more medially. The inferior wedge was designed to match the nipple to inframammary fold distance of the left breast, which was 8 cm in this case. The operation was performed under general anesthesia. The skin was incised along the medial side of the inferior pedicle design, and the pedicle was de-epithelialized medially around the nipple and inferiorly down to the inframammary fold (Fig 3). The surgical oncology team was then able to easily resect the specimen intact with this exposure, which weighed 286 g (Fig 4). The lateral breast and the nipple were rotated medially to meet the medial flap. The nipple was inset, and the T incision was closed using absorbable interrupted dermal sutures and a running subcuticular suture.

## RESULTS

**Figure 5:**
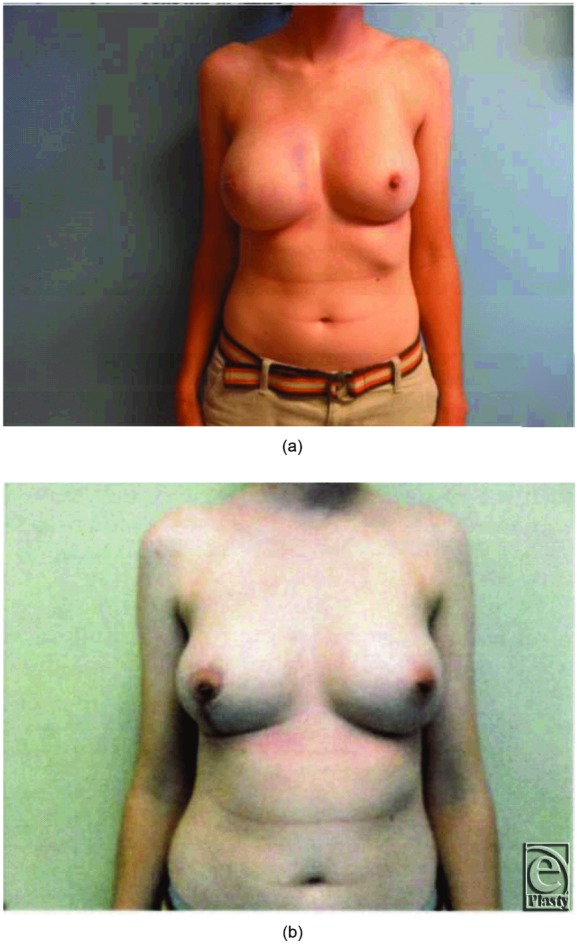
(a) Preoperative and (b) postoperative photographs.

At the completion of the procedure, the patient had excellent symmetry and the nipple had appropriate color and capillary refill. Final pathology was benign and showed fibroadenoma. At 3-month follow-up, the surgical site demonstrated well-healed incisions with preserved symmetry and a viable, sensate nipple-areolar complex (Fig 5).

## DISCUSSION

Fibroadenomas are the most common breast lesion in young women. Giant fibroadenomas are uncommon and make up 0.5% to 2% of all fibroadenomas.^5^ Although benign, they may be more clinically concerning due to size, progressive growth, associated pain, and psychological discomfort.^4^^,^^5^ Differential diagnosis should include a circumscribed fibrocystic mass, lipoma, giant hamartoma, cystosarcoma phyllodes, and other various carcinomas.^11^

It is essential to rule out the presence of malignancy, specifically phyllodes tumor. There have also been reports of rare cases describing adenocarcinoma and ductal carcinoma in situ arising within fibroadenomas.^12^ Fibroadenomas and phyllodes tumor have a similar clinical presentation, and unfortunately there are no imaging modalities that differentiate one from the other.^13^ One of the crucial steps in management is preoperative tissue diagnosis. Core needle biopsy is preferred over fine needle aspiration because fibroadenomas and phyllodes tumors have similar cytological features.^14^

Surgical excision is the standard treatment of giant fibroadenomas, but currently there are no clear guidelines regarding their surgical management. Multiple approaches have been reported in literature and include simple excision without reconstruction, various mastopexy, reduction, and augmentation techniques, and even subcutaneous mastectomy.^4^^,^^6^ It is possible that fibroadenomas of small to moderate size can be satisfactorily managed with enucleation; however, using this method to treat large tumors commonly leads to cosmetic sequelae that may require a secondary procedure.^14^

Oncoplastic surgical techniques were designed to address the issue of removing a tumor that was large relative to the size of the breast or removing a tumor that was located in a cosmetically unfavorable position. The 2 methods used for oncoplastic reconstruction are volume replacement and volume displacement.^9^ Volume replacement techniques provide the most benefit for women with small, nonptotic breasts. Different flaps can be used for reconstruction, such as the latissimus dorsi myocutaneous flap or pedicled perforator flaps, based on either the thoracodorsal or intercostal vessels.^15^ Volume displacement techniques are preferred for women with large, ptotic breasts. Volume displacement can be accomplished by various mastopexy or reduction mammoplasty techniques.^10^ Complementary symmetry procedures improve the patient's quality of life by reducing physical discomfort caused by resection of an abnormally large breast mass and the psychological burden of having noticeably asymmetric breasts.

Our patient had large breasts and was a good candidate for an oncoplastic volume displacement technique. Since the tumor was superior and medial to the nipple, we chose to use the inferior pedicle design of breast reduction and left the lateral portion of it intact to reconstruct the breast. Creating the incision for surgical oncology using the medial side of the Wise pattern permitted both adequate exposure for the resection of the tumor and the means to excise the redundant skin and redrape it over the remaining breast. The remaining defect was corrected using tissue from the inferior and lateral pole with reduction of the skin envelope. Patients often need to undergo reduction mammoplasty or mastopexy of the contralateral breast to achieve optimal symmetry. In this patient's case, the tumor itself was responsible for the volume difference and breast asymmetry. The remaining breast volume was normal and equal to the left, and no additional breast tissue was excised. This technique is effective for tumors of the upper pole of the breast and offers both a straightforward glandular resection without oncologic compromise and a dermoglandular pedicle that ensures reliable vascularity for the nipple-areolar complex.

In conclusion, oncoplastic breast reconstruction in a reduction pattern technique after giant fibroadenoma removal allows the application of plastic surgery principles for breast reduction without compromising the principles of surgical oncology. Oncoplastic surgery techniques have proven to be a reliable option in women undergoing breast conservation therapy for carcinoma of the breast and should be considered as a possible option when treating special therapeutic issues posed by benign breast disease.
